# Supplementary material for: Phenotypic Adaptation of Pseudomonas aeruginosa in the Presence of Siderophore-Antibiotic Conjugates during Epithelial Cell Infection
Source: Microorganisms. 2020 Nov 18;8(11):1820. doi: 10.3390/microorganisms8111820 (PMC7699141; doi:10.3390/microorganisms8111820)
Supplement: Supplementary file 1 [file microorganisms-08-01820-s001.pdf]

Supplemental materials

Phenotypic adaptation of *Pseudomonas aeruginosa* in the presence of siderophore-antibiotic conjugates during epithelial cell infection

Table S1. Strains and plasmids used in this study.

Strain	Collection ID	Relevant characteristics	Reference
Pseudomonas aeruginosa			
PAO1	PAO1	Wild-type strain	
ΔpvdFΔpchA	PAS283	PAO1; *pvdF* and *pchA* chromosomally deleted	[1]
ΔpvdFΔpchAΔpfeA	PAS294	PAO1; *pvdF, pchA* and *pfeA* chromosomally deleted	[2]
ΔpvdFΔpchAΔpirA	PAS348	PAO1; *pvdF, pchA* and *pirA* chromosomally deleted	[1]
ΔpvdFΔpchAΔpfeAΔpirA	PAS351	PAO1; *pvdF, pchA, pfeA* and *pirA* chromosomally deleted	[1]
ΔpvdFΔpchAΔfiuA	PAS534	PAO1; *pvdF, pchA* and *fiuA* chromosomally deleted	[3]
pfeEmcherry	PAS355	PAO1; *pfeEmcherry* chromosomally integrated	[4]

Table S2. Primers used for RT-qPCR analysis

Primer ID	Target	Sequence
uvrD F	*uvrD*	CTACGGTAGCGAGACCTACAACAA
uvrD R	*uvrD*	GCGGCTGACGGTATTGGA
GAPDH F	*GAPDH*	TGCACCACCAACTGCTTAGC
GAPDH R	*GAPDH*	GGCATGGACTGTGGTCATGAG
aprA F	*aprA*	AACCAGAAGATCAACCTCAACGA
aprA R	*aprA*	TCGACACATTGCCCTTCAAC
exoY F	*exoY*	AATGGATGGCGGAGCCTATA
exoY R	*exoY*	CAAGGCGTTGCCGAGAGAT
lasB F	*lasB*	CGCCTGGGCGAGAACA

lasB R	*lasB*	GGGAATCAGGTAGGAGACGTTGT
phzA2 F	*phzA2*	GGCACAACGTGCGGATCT
phzA2 R	*phzA2*	CGCACTCGACCCAGAAGTG
piv F	*piv*	GCGTGGGCCTGAAAACG
piv R	*piv*	CGATCCATTCGAGGGTTGTC
toxA F	*toxA*	CCCGGCGAAGCATGAC
toxA R	*toxA*	GGGAAATGCAGGCGATGA
amrZ F	*amrZ*	TCGCTCGCAGCCATCAC
amrZ R	*amrZ*	TCGAGTCGGGCGATGATC
fecA F	*fecA*	GATCGACGACCTGATCCTCAA
fecA R	*fecA*	GGTCATCGCCGAAAACGT
pirA F	*pirA*	GCCTGAACGCTTCCCAAA
pirA R	*pirA*	TGAAGGCCCGTGCGATA
pfeA F	*pfeA*	GCCGAGACCAGCGTGAAC
pfeA F	*pfeA*	GGCCGGATTCGATCTTGTT
fpvA F	*fpvA*	AGCCGCCTACCAGGATAAGC
fpvA R	*fpvA*	TGCCGTAATAGACGCTGGTTT
fptA F	*fptA*	GCGCCTGGGCTACAAGATC
fptA R	*fptA*	CCGTAGCGGTTGTTCCAGTT
foxA F	*foxA*	AAGGGCTCGGATACCCAGTT
foxA R	*foxA*	CGTTGGGATCGTGTTGCA
fiuA F	*fiuA*	GCCGCGACAAGAAGTTCAG
fiuA R	*fiuA*	ACGACTCCGCATAGGAGATATAGG
feoA F	*feoA*	CCTACCGCATCACCGGTTAT
feoA R	*feoA*	ACAGGCGTTGGCGATAGC
hasR F	*hasR*	AGCGCCTGCAGTTCAGCTA
hasR R	*hasR*	GTTCTCGGTGTTGAGCATGTTG
phuR F	*phuR*	GGTCGAACTGCCCAACGA
phuR R	*phuR*	TACGATGTCCGGATCGACGTA

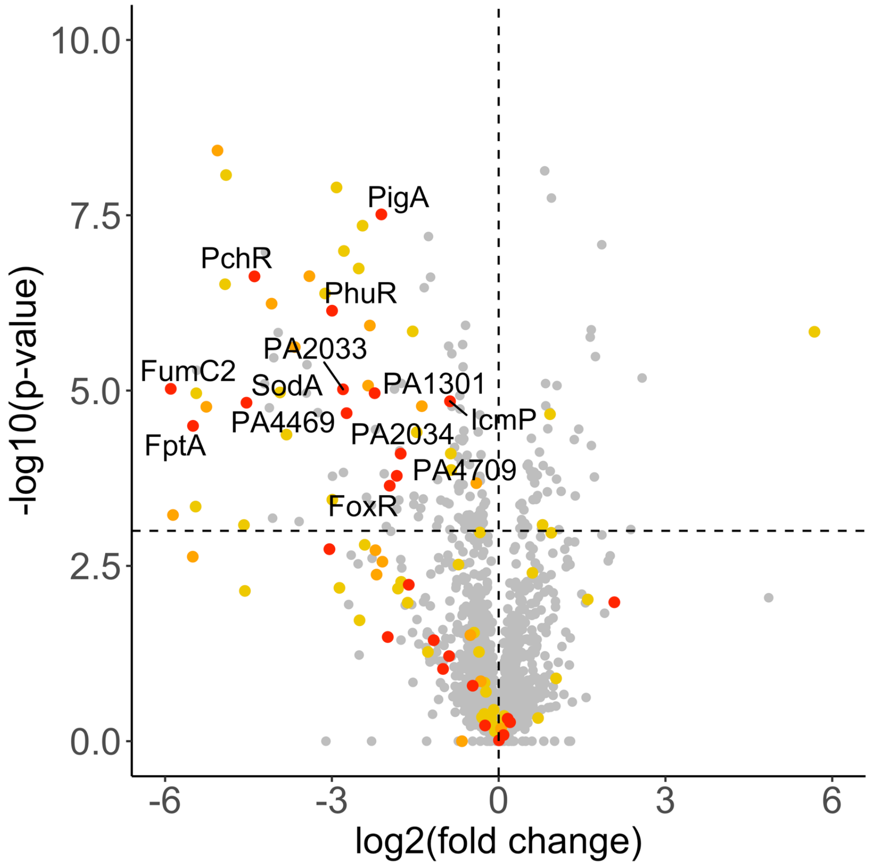

Figure S1. Differential production of iron-regulated proteins in the presence of ALBO based on the Palma, Worgall, and Quadri (2003) and Ochsner et al. (2002) studies [5,6]. Both studies were transcriptome analyses of the *P. aeruginosa* response to iron. The analysis presented here was performed on *P. aeruginosa* PAO1 cells after a 3-h infection of A549 epithelial cells in RPMI medium, in the presence or absence of 10 µM ALBO. The yellow points correspond to proteins identified in one of the two analyses, the orange points to proteins identified in the two analyses, and the red points to proteins belonging to the direct Fur regulon, as described in the Collectf database.

Synthesis of TCVL6.

General informations

TCVL6 was synthesized as described in 1S Scheme. Linezolid was purchased from Sigma-Aldrich. Protected TCV **1** and linezolid-azide derivative **2** were synthesized according previously described protocols [7,8]. All reactions were carried out under argon (technical

quality, Air products). Solvents used were of analytical grade purity (>99.9%). When necessary, solvents and bases were purchased extra-dry. All other chemicals were obtained from commercial suppliers and were used as received, unless otherwise stated. All reactions were monitored by thin-layer chromatography (TLC) using *Merck* precoated silica gel $60F^{254}$ (0.25 mm). TLC are visualized using UV (254 nm/365 nm, *Vilber Lourmat*, VL-4LC) and/or using classical revelation mixtures (sulfuric vaniline, potassium permanganate, ninhydrin reagent). Final conjugate **3** was also detected on TLC using a 2% hydromethanolic solution of $FeCl_3$ (2%). Before chromatographic purification reaction mixtures were adsorbed on silica gel (60-200 µm, VWR Chemicals). Chromatographic purifications were performed on a *Reveleris* (*Grace Davison Discovery Sciences*) purification device using PuriFlash® pre-packed silica gel columns (Interchim, Montluçon, France). NMR spectra were recorded on Bruker Avance 400 (^{1}H: 400 MHz, ^{13}C: 100 MHz, ^{19}F : 376 MHz) or Avance 500 (^{1}H: 500 MHz, ^{13}C: 125 MHz, ^{19}F : 470 MHz), using the residual non-deuterated solvent as reference. The chemical shifts (δ) and coupling constants (J) are expressed in ppm and hertz respectively. Multiplicity is indicated as follow: s for singlet, d for doublet, t for triplet, q for quadruplet, quint for quintuplet and m for a multiplet. The letter "b" before the multiplicity indicate a broaden signal. Mass spectra were recorded in the *Service Commun d'Analyses* (SCA) *de la Faculté de Pharmacie de l'Université de Strasbourg* and were measured in ES-TOF experiments on a *Bruker Daltonic* MicroTOF mass spectrometer. LC/HRMS were measured after calibration on an *Agilent* QToF.

Protocol

Linezolid azide **2** (1 eq.) and alkyne-vector **1** (1,2 eq.) were solubilized in THF (final concentration 0.1 M). An aqueous solution of $CuSO_4$ (200 mg/mL, 1 eq.) and sodium ascorbate (5 eq.) were successively added [9]. The mixture was sonicated and further stirred at 20°C

under argon till the total consumption of limiting reagent (linezolid azide). The mixture was then filtered through a celite pad and the filtrate was adsorbed on silical gel. The crude mixture was then purified on silica gel using a gradient of EtOH in CH_2Cl_2. The resulting white solid was dissolved in the mixture CH_2Cl_2/TFA/Triisopropylsilane/EtOH (70/20/5/5, 5 mL for 0.1 mmol of starting linezolid azide **2**). The solution was stirred at 20°C till the total consumption of the starting material. Solvents were then evaporated from the mixture under reduced pressure. The oily residue was dissolved in a minimum volume of THF then cyclohexane was added dropwise till the precipitation of the expected conjugate as a thin powder. The pure conjugate was filtered off on a Hirsch funnel.

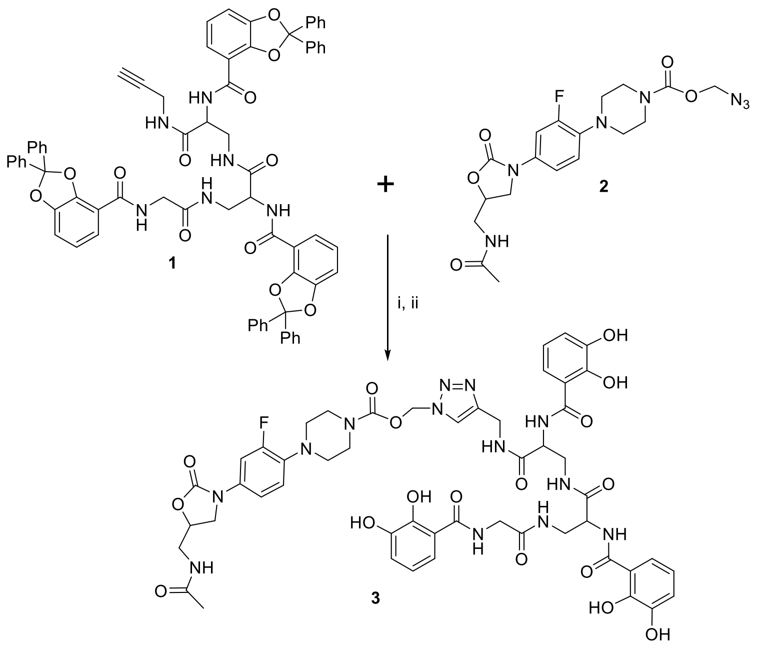

Scheme S1: Synthesis of TCVL6 (3). i. $CuSO_4$, sodium ascorbate, THF/H_2O, (((, 20°C. ii. CH_2Cl_2/TFA/Triisopropylsilane/EtOH (70/20/5/5, 20°C.

Spectroscopic analysis of TCVL6.

^{1}H NMR (500 MHz, DMSO-d_6): δ 11.98 (s, 1H), 11.73 (m, 2H), 9.05 (br s, 3H), 8.79 (s, 1H), 8.56 (d, J = 6.9 Hz, 1H), 8.53 (d, J = 6.9 Hz, 1H), 8.45 (t, J = 5.5 Hz, 1H), 8.18 (t, J = 5.5 Hz, 1H), 8.02 (t, J = 5.5 Hz, 1H), 7.93 (t, J = 5.5 Hz, 1H), 7.81 (s, 1H), 7.46 (t, J = 7.2 Hz, 1H), 7.28-7.22 (m, 1H), 7.08-6.99 (m, 3H), 6.95-6.89 (m, 1H), 6.84-6.78 (m, 1H), 6.72-6.66 (m, 3H), 6.49-6.40 (m, 3H), 6.03 (s, 2H), 4.35-4.28 (m, 1H), 4.19-4.08 (m, 2H), 4.02-3.91 (m, 2H), 3.89-3.85 (m, 1H), 3.71-3.65 (m, 1H), 3.55-2.91 (m, 14H), 1.44 (s, 3H). **^{19}F NMR** (125 MHz, DMSO-d_6): δ -73.72, -121.19. **^{13}C NMR** (125 MHz, DMSO-d_6): δ 169.7, 169.6, 169.5, 169.4, 169.3, 169.2, 169.1, 162.5, 149.2, 149.0, 146.1, 146.0, 145.4, 137.1, 132.8, 129.7, 128.7, 124.1, 119.9, 118.9, 118.8, 118.2, 118.1, 117.8, 117.7, 115.5, 114.1, 71.6, 68.4, 53.9, 53.8, 53.7, 50.1, 47.3, 47.2, 43.7, 42.4, 41.5, 10.5, 40.3, 40.2, 40.1, 35.9, 30.9, 27.5, 23.4, 23.3, 22.5, 21.8, 21.7. **HRMS:** $C_{50}H_{54}FN_{13}O_{17}$: calcd: 1127.37447, found: 1127.37501.

LC-HRMS Spectra of compound 3 (TCVL6)

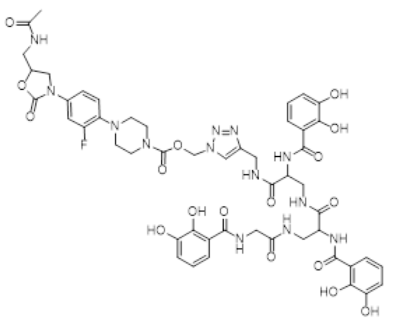

Chemical Formula: $C_{50}H_{54}FN_{13}O_{17}$
Exact Mass: 1127,37447
Molecular Weight: 1128,05440

**Data Filename**	QT11799.d	**Sample Name**	NZ108
**Inj. Vol.**	0.1	**Position**	P1-B3
**Instrument Name**	SCA Illkirch QToF	**User Name**	PW
**Acq Method**	C18-2,1x5x1,8DMSO.m	**Acquired Time**	2/4/2016 12:07:19 PM
**IRM Calibration Status**	Success	**DA Method**	C18-2,1x5x1,8.m
**Comment**			

Sample Group **Info.**

User Chromatograms

Fragmentor Voltage 120 **Collision Energy** 0 **Ionization Mode** ESI

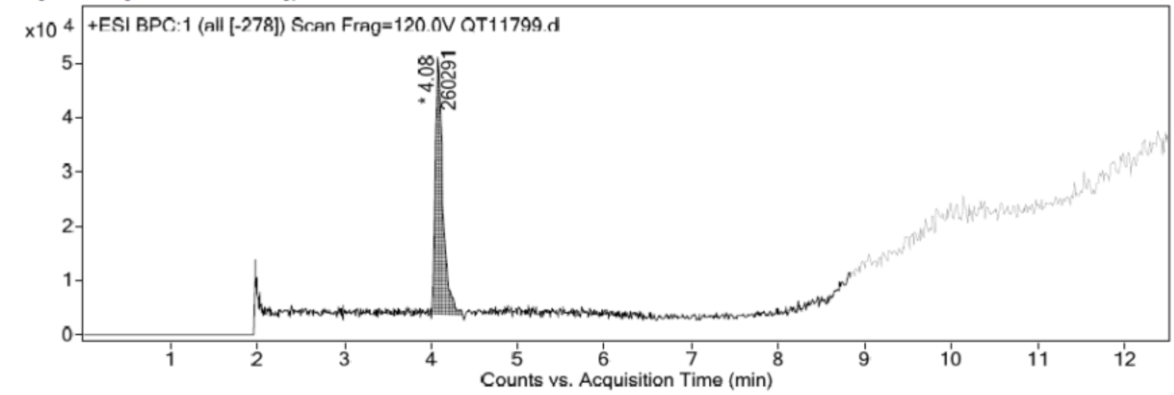

Integration Peak List

Start	RT	End	Height	Area	Area %	AreaSum%
3.97	4.08	4.37	47512	260291	100	100

Spectrum Source	Fragmentor Voltage	Collision Energy	Ionization Mode
Peak (1) in "+ BPC:1 (all [-278]) Scan"	120	0	ESI

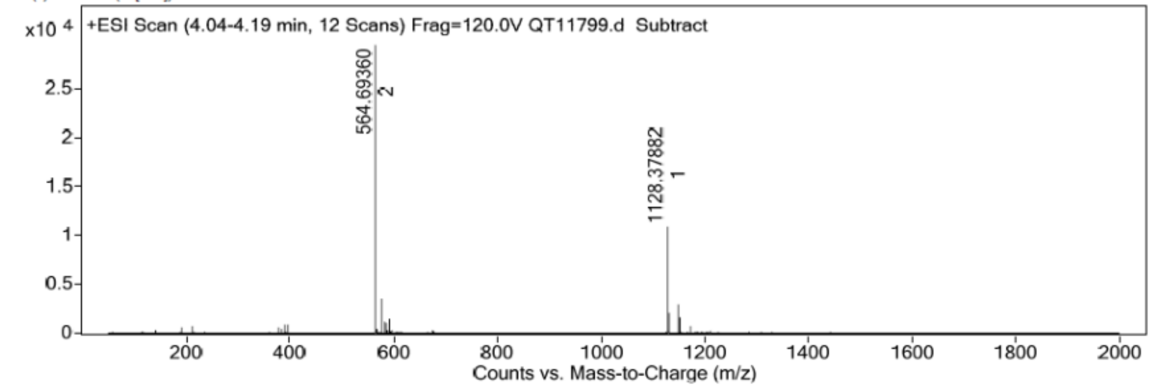

Peak List

m/z	z	Abund	Formula	Ion
564.6936	2	29418.6	C50 H56 F N13 O17	(M+2H)+2
565.19508	2	17423.8	C50 H56 F N13 O17	(M+2H)+2
565.69599	2	6106.5	C50 H56 F N13 O17	(M+2H)+2
575.68361	2	3582.9		
576.18663	2	2021		
1128.37882	1	10820.6	C50 H55 F N13 O17	(M+H)+
1129.38218	1	6280.4	C50 H55 F N13 O17	(M+H)+
1130.38535	1	2133	C50 H55 F N13 O17	(M+H)+
1150.36048	1	2936.7	C50 H54 F N13 Na O17	(M+Na)+
1151.36413	1	1682.2	C50 H54 F N13 Na O17	(M+Na)+

Formula Calculator Results

Formula	Best	Mass	Tgt Mass	Diff (ppm)	Mz	Ion Species	Score
C46 H50 F N19 O15		1127.37167	1127.37178	0.1	1128.37882	C46 H51 F N19 O15	99.57
C49 H49 N19 O14		1127.37166	1127.37064	-0.91	1128.37882	C49 H50 N19 O14	98.92
C45 H54 F N15 O19		1127.37165	1127.37044	-1.07	1128.37882	C45 H55 F N15 O19	98.77
C62 H50 F N11 O10		1127.3716	1127.37262	0.9	1128.37882	C62 H51 F N11 O10	98.03
C53 H53 N13 O16		1127.37162	1127.37332	1.51	1128.37882	C53 H54 N13 O16	97.9
C48 H53 N15 O18		1127.37164	1127.3693	-2.08	1128.37882	C48 H54 N15 O18	96.54
C50 H54 F N13 O17	TRUE	1127.37163	1127.37447	2.52	1128.37882	C50 H55 F N13 O17	95.18
C54 H49 N17 O12		1127.37164	1127.37466	2.68	1128.37882	C54 H50 N17 O12	94.19
C57 H50 F N13 O12		1127.37162	1127.36859	-2.68	1128.37882	C57 H51 F N13 O12	94.08
C51 H50 F N17 O13		1127.37165	1127.3758	3.69	1128.37882	C51 H51 F N17 O13	90.37
C46 H50 F N19 O15		1127.37139	1127.37178	0.34	1150.36048	C46 H50 F N19 Na O15	98.44
C49 H49 N19 O14		1127.37139	1127.37064	-0.66	1150.36048	C49 H49 N19 Na O14	98.12
C45 H54 F N15 O19		1127.37137	1127.37044	-0.82	1150.36048	C45 H54 F N15 Na O19	98.11
C48 H53 N15 O18		1127.37136	1127.3693	-1.83	1150.36048	C48 H53 N15 Na O18	96.2
C62 H50 F N11 O10		1127.37132	1127.37262	1.15	1150.36048	C62 H50 F N11 Na O10	96.17
C53 H53 N13 O16		1127.37134	1127.37332	1.76	1150.36048	C53 H53 N13 Na O16	96.08
C57 H50 F N13 O12		1127.37134	1127.36859	-2.43	1150.36048	C57 H50 F N13 Na O12	93.62
C50 H54 F N13 O17	TRUE	1127.37135	1127.37447	2.77	1150.36048	C50 H54 F N13 Na O17	93.18
C54 H49 N17 O12		1127.37136	1127.37466	2.92	1150.36048	C54 H49 N17 Na O12	92.17
C60 H49 N13 O11		1127.37133	1127.36745	-3.45	1150.36048	C60 H49 N13 Na O11	89.46
C53 H53 N13 O16		1127.37264	1127.37332	0.6	564.6936	C53 H55 N13 O16	99.64
C46 H50 F N19 O15		1127.3727	1127.37178	-0.81	564.6936	C46 H52 F N19 O15	99.61
C62 H50 F N11 O10		1127.37262	1127.37262	-0.01	564.6936	C62 H52 F N11 O10	98.85
C50 H54 F N13 O17	TRUE	1127.37265	1127.37447	1.61	564.6936	C50 H56 F N13 O17	98.67
C49 H49 N19 O14		1127.37269	1127.37064	-1.82	564.6936	C49 H51 N19 O14	98.32
C54 H49 N17 O12		1127.37267	1127.37466	1.77	564.6936	C54 H51 N17 O12	98.14
C45 H54 F N15 O19		1127.37267	1127.37044	-1.98	564.6936	C45 H56 F N15 O19	97.96
C51 H50 F N17 O13		1127.37267	1127.3758	2.78	564.6936	C51 H52 F N17 O13	96.21
C48 H53 N15 O18		1127.37267	1127.3693	-2.99	564.6936	C48 H55 N15 O18	95.76
C42 H53 N19 O19		1127.37271	1127.37651	3.37	564.6936	C42 H55 N19 O19	94.43

References

1. Gasser, V.; Baco, E.; Cunrath, O.; August, P.S.; Perraud, Q.; Zill, N.; Schleberger, C.; Schmidt, A.; Paulen, A.; Bumann, D.; et al. Catechol siderophores repress the pyochelin pathway and activate the enterobactin pathway in *Pseudomonas aeruginosa*: an opportunity for siderophore-antibiotic conjugates development. *Environ. Microbiol.* **2016**, *18*, 819–832, doi:10.1111/1462-2920.13199.
2. Paulen, A.; Gasser, V.; Hoegy, F.; Perraud, Q.; Pesset, B.; Schalk, I.J.; Mislin, G.L.A. Synthesis and antibiotic activity of oxazolidinone-catechol conjugates against *Pseudomonas aeruginosa*. *Org. Biomol. Chem.* **2015**, *13*, 11567–11579, doi:10.1039/c5ob01859e.
3. Normant, V.P.; Josts, I.; Kuhn, L.; Perraud, Q.; Fritsch, S.; Hammann, P.; Mislin, G.L.A.; Tidow, H.; Schalk, I.J. Nocardamine-dependent iron uptake in *Pseudomonas aeruginosa*: exclusive involvement of the FoxA outer membrane transporter. *ACS Chem. Biol.* **2020**, 15, 2741-2751. doi: 10.1021/acschembio.0c00535. .
4. Perraud, Q.; Moynié, L.; Gasser, V.; Munier, M.; Godet, J.; Hoegy, F.; Mély, Y.; Mislin, G.L.A.; Naismith, J.H.; Schalk, I.J. A Key Role for the Periplasmic PfeE Esterase in Iron Acquisition via the Siderophore Enterobactin in *Pseudomonas aeruginosa*. *ACS Chem. Biol. 2018*, *13*, 2603–2614, doi:10.1021/acschembio.8b00543.
5. Palma, M.; Worgall, S.; Quadri, L.E.N. Transcriptome analysis of the Pseudomonas aeruginosa response to iron. *Arch. Microbiol.* **2003**, *180*, 374–379, doi:10.1007/s00203-003-0602-z.

6. Ochsner, U.A.; Wilderman, P.J.; Vasil, A.I.; Vasil, M.L. GeneChip expression analysis of the iron starvation response in *Pseudomonas aeruginosa*: identification of novel pyoverdine biosynthesis genes. *Mol. Microbiol.* **2002**, *45*, 1277–87.
7. Baco, E.; Hoegy, F.; Schalk, I.J.; Mislin, G.L. Diphenyl-benzo[1,3]dioxole-4-carboxylic acid pentafluorophenyl ester: a convenient catechol precursor in the synthesis of siderophore vectors suitable for antibiotic Trojan horse strategies. *Org. Biomol. Chem.* **2014**, *12*, 749–57, doi:10.1039/c3ob41990h.
8. Paulen, A.; Hoegy, F.; Roche, B.; Schalk, I.J.; Mislin, G.L.A. Synthesis of conjugates between oxazolidinone antibiotics and a pyochelin analogue. *Bioorg. Med. Chem. Lett.* **2017**, *27*, 4867–4870, doi:10.1016/j.bmcl.2017.09.039.
9. Rostovtsev, V.V.; Green, L.G.; Fokin, V.V.; Sharpless, K.B. A Stepwise Huisgen Cycloaddition Process: Copper(I)-Catalyzed Regioselective "Ligation" of Azides and Terminal Alkynes. *Ang. Chem. Int. Ed.* **2002**, *41*, 2596–2599, doi:10.1002/1521-3773(20020715)41:14<2596::AID-ANIE2596>3.0.CO;2-4.
